# Integrative GWAS and transcriptomic analyses reveal markers and candidate genes associated with resistance to *Botrytis cinerea* fruit rot in blueberry

**DOI:** 10.1093/hr/uhag092

**Published:** 2026-03-13

**Authors:** Lushan Ghimire, Yichun Wang, Paul Adunola, Wardatou Boukari, Gonzalo Casorzo, Felix Enciso-Rodriguez, Philip F Harmon, Juliana Benevenuto, Patricio R Munoz

**Affiliations:** Blueberry Breeding and Genomics Lab, Horticultural Sciences Department, University of Florida, Gainesville, FL 32611, USA; Blueberry Breeding and Genomics Lab, Horticultural Sciences Department, University of Florida, Gainesville, FL 32611, USA; Blueberry Breeding and Genomics Lab, Horticultural Sciences Department, University of Florida, Gainesville, FL 32611, USA; Plant Pathology Department, University of Florida, Gainesville, FL 32611, USA; Blueberry Breeding and Genomics Lab, Horticultural Sciences Department, University of Florida, Gainesville, FL 32611, USA; Blueberry Breeding and Genomics Lab, Horticultural Sciences Department, University of Florida, Gainesville, FL 32611, USA; Plant Pathology Department, University of Florida, Gainesville, FL 32611, USA; Blueberry Breeding and Genomics Lab, Horticultural Sciences Department, University of Florida, Gainesville, FL 32611, USA; Blueberry Breeding and Genomics Lab, Horticultural Sciences Department, University of Florida, Gainesville, FL 32611, USA

## Abstract

*Botrytis cinerea* is a broad host range fungal pathogen causing gray mold disease and crop losses worldwide. In blueberries, symptoms include blossom blight in the field and postharvest fruit rot, affecting the entire supply chain. With control options constrained by regulatory restrictions and fungicide resistance, the dissection of the genetic and molecular basis of blueberry response to *B. cinerea* can accelerate breeding for resistance. In this study, we phenotyped 354 blueberry selections using a high-throughput *Botrytis* infection fruit assay. The same population was genotyped by targeted sequencing for genome-wide association study (GWAS). In addition, we performed RNA-seq time-course (0–96 hours post-inoculation) for resistant and susceptible genotypes. Our results showed a continuum of tolerance levels and moderate narrow-sense heritability estimates for the disease-related traits (0.46–0.61). GWAS identified small-effect loci, consistent with quantitative resistance observed in other host plant species. Intersecting differentially expressed genes with GWAS intervals revealed eight candidate genes. Transcriptomic analyses showed that, at early stages, the resistant genotype upregulated components of basal innate immunity, including wax and cutin biosynthesis, responses to wounding and fungal-derived molecules, the MAPK cascade, and ethylene and jasmonate signaling. In contrast, susceptible genotypes displayed delayed activation of these defense pathways and altered cell wall-related processes. The moderate correlation between disease traits and wax bloom further supported a role for wax in disease response. Together, our findings provide molecular markers and candidate genes for *Botrytis* fruit rot resistance in blueberry with significant applications in breeding programs and opportunities to future validation studies.

## Introduction

*Botrytis cinerea* is necrotrophic fungal pathogen causing gray mold disease in more than a thousand plant species. It is considered one of the major causes of preharvest and postharvest losses in several important crops, including strawberry, tomato, grape, and blueberry, among others, with global economic losses estimated at up to $10 billion annually [1–3]. In blueberries (*Vaccinium corymbosum* and hybrids), it causes blossom blight and fruit rot, resulting in flower drop, reduced fruit set, or shortened postharvest life [4–6]. Outbreaks reported in blueberry reduced yield by 30%–40% and degrade fruit quality during storage and transport [7, 8]. These losses jeopardize the industry’s continued expansion into international markets, where shipments may require up to 25 days in cold storage depending on the origin and destination countries. Blueberries have seen a rapid increase in production, with a 142% rise over the past decade, primarily for the fresh market. As industry seeks to extend storage life to enhance marketing periods and boost exports, postharvest diseases like gray mold become critical constraints [9].

Gray mold decay in blueberry can arise from natural contamination during flowering and fruit development under field conditions or during postharvest fruit handling. *Botrytis cinerea* persists as sclerotia or conidia and can remain quiescent in plant tissues until high humidity (>95%) and moderate temperatures (15°C–20°C) trigger germination and infection [5]. The disease cycle begins with sporulation on infected tissue, and conidia dispersion by wind and water [10]. Frost-delayed corolla drops increase vulnerability to infection, which can progress from the corolla to the ovary, peduncle, and entire berry cluster. Latent infections in young fruit can later express as wrinkling, atrophy, and severe rot [11, 12]. Symptoms can appear even under refrigeration at 0°C, which is usually the commercial fruit storage and shipment temperature [9, 13]. Although fungicides are widely used for control, increasing resistance to their active ingredients and regulatory limits underscore the urgent need for durable genetic solutions [14, 15].

Upon entry, *B. cinerea* deploys toxins and cell-wall–degrading enzymes that promote tissue necrosis and colonization [16, 17]. Plant defense therefore hinges on physical barriers and inducible responses, such as the cuticle (waxes/cutin) that limits adhesion and penetration, and the plant cell wall that provides a structural barrier [18–20]. Unlike many biotrophic pathogens, *B. cinerea* seldom triggers classical effector-triggered immunity and can manipulate host cell death [21]. Instead, effective resistance has been more related to the timing and intensity of pattern-triggered immunity processes (such as hormone signaling, control of reactive oxygen species—ROS, secondary metabolism) rather than on a single effector–recognition event [22–25]. Consistent with this view, transcriptomic studies in tomato, grape, and Arabidopsis have shown that more resistant genotypes reinforce structural barriers and tune ROS/secondary metabolism earlier than susceptible ones [16, 26, 27].

To identify quantitative trait locus (QTL) linked to *Botrytis* resistance, marker-trait associations studies have been performed in several plant species, such as in Arabidopsis [28–31], tomato [32, 33], grape [26], strawberry [34], *Brassica rapa* [35], chickpea [36], and Gerbera [37]. In all cases, multiple small-to-medium-effect QTL were identified governing susceptibility to *B. cinerea*. Transcriptomic analyses have also been performed to narrow down candidate genes in associated genomic regions [26], detect expression QTLs [30], and genotype-specific defense responses [38, 39]. Despite advances in molecular breeding for gray mold resistance in other crop species, the extent of the phenotypic diversity, the genetic architecture underlying its variation, and the molecular mechanisms of *B. cinerea* response in contrasting blueberry genotypes remain unexplored.

To address this gap, we first performed a high-throughput fruit inoculation assay for 354 southern highbush blueberry genotypes. Genome wide association study (GWAS) was performed using single nucleotide polymorphisms (SNPs) from targeted sequencing technology. Then, resistant and susceptible genotypes were selected for a comparative transcriptome analysis during *B. cinerea* fruit infection. This study provides critical genomic resources for blueberry breeding by nominating markers and candidate genes for *B. cinerea* resistance in blueberries.

## Results

### Phenotypic analysis

**Figure 1:**
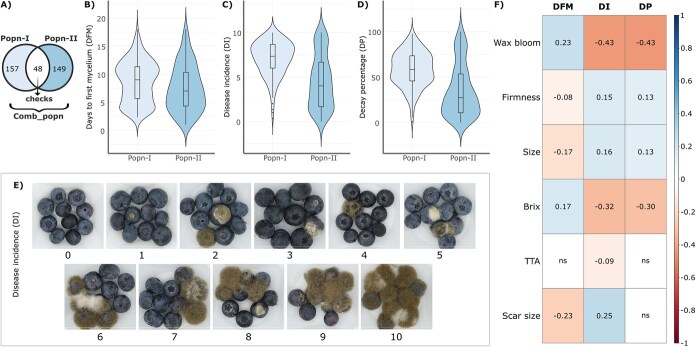
Phenotypic response exhibited by blueberry genotypes after inoculation with *B. cinerea*. (A) Combination (Comb_popn) of two blueberry breeding populations (Popn-I and Popn-II) and common checks used in this study. Violin plot showing the distribution of (B) DFM, (C) DI, and (D) fruit DP. (E) Spectrum of DI across genotypes after 16 dpi inoculation with *B. cinerea,* where 0 means no visible symptoms in any berry, and 10 means visible symptoms in all berries*.* (F) Heatmap of Pearson correlation coefficients (*r*) between disease- and fruit quality-related traits. Significant correlations are highlighted in the color scale, while non-significant correlations are displayed as ns.

In this study, we assessed disease severity following inoculation with *B. cinerea* in 354 southern highbush blueberry genotypes derived from two breeding cycle populations (Popn-I and Popn-II) and assessed in two different years (2022 and 2023, respectively). A total of 48 genotypes were used as checks across years (Fig. 1A). Using a high-throughput screening protocol (Fig. S1), we recorded the days to the appearance of first mycelium (DFM), the disease incidence (DI) as the counts of infected berries within a clamshell, and the fruit decay percentage (DP) as the spread of mycelia on the fruit surface (DP). Correlation analysis across the 48 overlapping checks yielded a coefficient of 0.67.

We observed substantial phenotypic variation for the three traits: DFM, DI, and DP (Fig. 1B–E). DFM was negatively correlated with both DI and DP (*r* = −0.74), whereas DI and DP were strongly positively correlated (*r* = 0.97). Mycelial growth was detected as early as 1 day post-inoculation (dpi) in both populations. By 16 dpi, three genotypes in Popn-II showed no mycelium across all three replicates, and five genotypes in Popn-I showed no mycelium in at least one replicate. At the opposite side, 12 genotypes in Popn-I and 11 genotypes in Popn-II had at least one replicate with all 10 berries per clamshell infected and fully decayed (10 DI and 100% DP).

### Phenotypic correlation analysis

Following surface inoculation, *B. cinerea* can penetrate the skin layers, colonizes sub-epidermal tissues, and ultimately spreads throughout the flesh of susceptible fruits. Therefore, we reasoned that external and internal fruit quality traits could be biologically relevant for disease progression. Pearson correlations were calculated between fruit-quality traits and the three disease-traits assessed (DFM, DI, and DP), where higher DFM indicates greater resistance and higher DI/DP indicate greater susceptibility (Fig. 1F). Wax bloom stood out as it showed a moderate negative correlation with both DI and DP (*r* = −0.43), indicating that greater wax coverage was associated with delayed mycelial onset and reduced incidence/decay. Soluble solids content (°Brix) also showed a moderate negative correlation with DI and DP (*r* = 0.32). Other fruit quality-related traits (firmness, berry size, scar size, and acidity) were either non-significant or had very small correlation coefficients, indicating weak linear associations between these variables and disease-related traits.

### Genetic parameters

For genetic parameter estimation and downstream analyses, we pooled the two populations into a combined dataset (Comb_popn) after adjusting for year effect. Across all three traits, the additive variance component (σ_a_^2^) exceeded the non-additive component (σ_d_^2^). Narrow-sense heritability (*h^2^*) ranged from 0.46 to 0.61, and broad-sense heritability (*H^2^*) from 0.68 to 0.77, with DFM showing the lowest and DI the highest values (Table 1).

**Table 1:** Estimates of additive (${\sigma}_a^2$**)** and non-additive (${\sigma}_d^2$**)** variance components, narrow (*h^2^*) and broad sense (*H^2^*) heritabilities with their SD in the combined blueberry population (Comb_popn) for DFM, DI, and fruit DP 16 dpi with *B. cinerea*.

**Trait**	${\sigma}_a^2$ **± SD**	${\sigma}_d^2$ **± SD**	***h*** ^***2***^ **± SD**	***H*** ^***2***^ **± SD**
DFM	7.55 ± 1.46	3.57 ± 1.13	0.46 ± 0.07	0.68 ± 0.07
DI	4.39 ± 0.56	1.13 ± 0.27	0.61 ± 0.05	0.77 ± 0.04
DP	351.73 ± 47.11	99.99 ± 24.92	0.58 ± 0.05	0.75 ± 0.05

### Genome-wide association analysis

**Figure 2:**
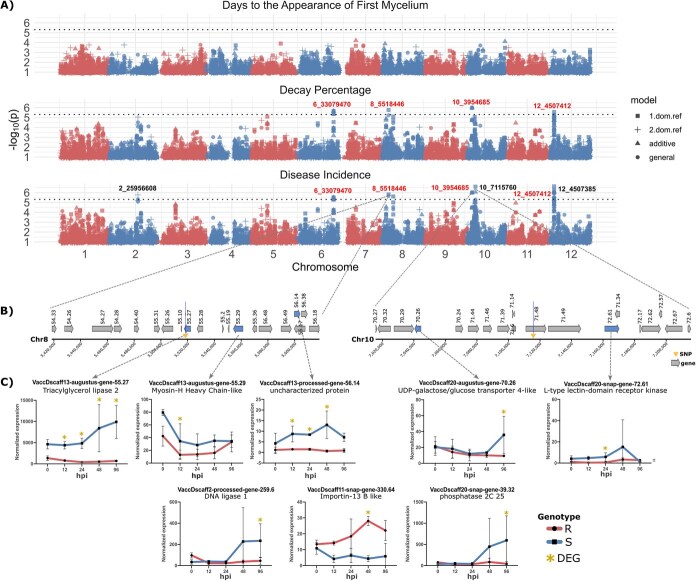
Significant associated genomic regions and expression of candidate genes related to *Botrytis* resistance in blueberry fruits. (A) Manhattan plots showing the association of SNP markers with three disease-related traits against *B. cinerea* in the combined southern highbush blueberry population (Comb_popn). Adjusted Bonferroni correction was used to establish a *P*-value detection threshold for statistical significance (dotted lines). Significant SNPs consistent across two traits are highlighted in red font. (B) Genes within a genomic window of ±100 kb flanking significant SNPs at chromosome 8 and chromosome 10, highlighting genes differentially expressed between resistant and susceptible genotypes during *Botrytis* infection (blue arrows). (C) Expression profiles of eight candidate genes within the GWAS regions that were statistically significant in at least one timepoint comparison (12–96 hpi) during the *Botrytis* infection between resistant (red) and susceptible (blue) genotypes in a transcriptomics experiment. Asterisks indicate DEGs identified between genotypes at each time point using DESeq2-adjusted *P*-values. More details about the transcriptomics analyses are provided in the subsequent sections.

A total of 38 379 SNPs, mapped to the 12-haploid chromosome-scale scaffolds of the blueberry genome (Fig. S2), were tested for association with *B. cinerea* response related traits. GWAS analyses controlling for population structure and kinship were performed for several gene action models. After combining both populations and adjusting phenotypes for year effect, we observed no significant associations for DFM (Fig. 2A). In contrast, four significant associations were identified for DP in chromosomes 6, 8, 10, and 12 (Fig. 2A, Fig. S3). Each explained a small fraction of the phenotypic variance, and no major-effect QTL was identified (Table 2).

**Table 2:** Significant SNPs associated with *B. cinerea* response in southern highbush blueberries for DI and fruit DP.

**Trait**	**Chrom**	**Position**	**Model**	***P*-value**	**MAF**	**Effect**	**Threshold**	**Score**	**PVE**	**Ref/Alt**
DI	2	25 956 608	General 2-dom-ref	0.00 0.00	0.03	NA 3.86	5.31 5.30	5.32 5.77	6.80 4.80	T/A
DI	6	33 079 470	General Additive 1-dom-ref	0.19 0.00 0.03	0.02	NA 3.74 3.74	5.31 5.31 5.28	5.51 5.51 5.51	0.50 12.60 1.40	T/A
DI	8	5 518 446	General 1-dom-ref	0.47 0.04	0.29	NA, 3.33	5.31 5.28	5.67 5.82	0.40 1.20	A/G
DI	10	3 954 685	general	0.29	0.04	NA	5.31	6.00	0.30	C/A
DI	10	7 115 760	Additive 1-dom-ref	0.00 0.00	0.12	0.94 1.98	5.31 5.28	6.05 6.60	4.10 4.50	C/G
DI	12	4 507 412	general	0.00	0.05	NA	5.31	6.00	2.30	G/A
DI	12	4 507 385	additive	0.00	0.04	1.81	5.31	6.10	3.40	T/C
DP	6	33 079 470	General Additive 1-dom-ref	0.06 0.06 0.05	0.02	NA 29.25 29.25	5.31 5.31 5.28	5.66 5.66 5.66	1.00 1.00 1.10	T/A
DP	8	5 518 446	1-dom-ref	0.74	0.29	29.89	5.28	5.77	0.00	A/G
DP	10	3 954 685	General Additive 1-dom-ref	0.01 0.01 0.57	0.04	NA 33.94 33.94	5.31 5.31 5.28	5.98 5.98 5.98	2.00 2.00 0.10	C/A
DP	12	4 507 412	1-dom-ref	0.00	0.05	19.99	5.28	5.57	2.50	G/A

The corresponding SNP location, gene action model, *P*-value, MAF, effect, adjusted Bonferroni threshold, score (i.e. −log10(*P*-value)), PVE, and reference (Ref) and alternative (Alt) allele are reported.

For DI, seven significant SNP associations were identified across chromosomes 2, 6, 8, 10 and 12 (Fig. 2A, Fig. S3). The strongest association was found on chromosome 6, accounting for 12.6% of the additive phenotypic variance, with other loci contributing smaller proportions (Table 2). Notably, four of the seven DI loci were also identified for DP.

### Differentially expressed genes

**Figure 3:**
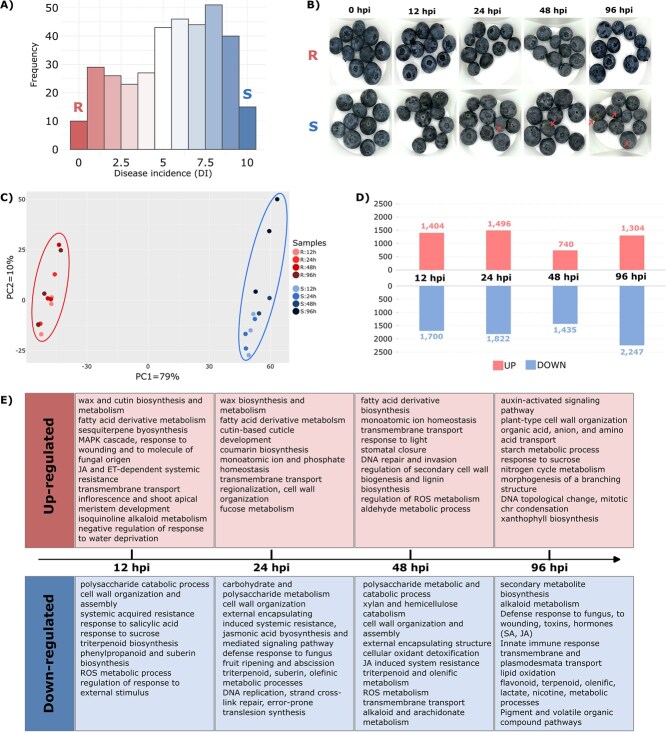
Transcriptomics experiment and expression analyses. (A) DI histogram from the screening assay, illustrating selection of resistant-R (low DI) and susceptible-S (high DI) genotypes from the tail ends of the spectrum. (B) Resistant and susceptible blueberry fruits at 0, 12, 24, 48, and 96 hpi, with arrows pointing to visible symptoms. (C) PCA of whole gene expression across all samples. Each point represents one biological replicate, colored by genotype (R or S) and time point. (D) Number of DEGs identified between R and S genotypes at each time point after removal of DEGs detected at 0 hpi. Red and blue bars indicate up- and down-regulated genes in the resistant genotype relative to the susceptible genotype. (E) Summary of relevant enriched GO BP terms for up- (red) and down-regulated (blue) genes in the comparison of resistant (R) against susceptible (S) genotypes from 12 to 96 hpi. Full GO term list and descriptions are provided in Table S2.

To contextualize the GWAS signals and understand the molecular mechanisms of host responses, we performed a time-course RNA-seq analysis. We selected one resistant (R) and one susceptible (S) genotype from opposite ends of the phenotypic distribution for DI (Fig. 3A). Ripe fruits were harvested and sampled for non-inoculated fruits (0 hours post inoculation, hpi) and inoculated fruit at 12, 24, 48, and 96 hpi. Symptoms appeared in S at 24 hpi, whereas R remained asymptomatic until the end of the experiment (Fig. 3B). To assess overall variation in gene expression patterns, we performed a principal component analysis (PCA), which revealed a clear separation between the two genotypes along the first principal component (PC1), explaining 79% of the total variance (Fig. 3C).

Differentially expressed genes (DEGs) were detected at every time point via the R vs S comparison and excluding the DEGs between non-inoculated fruits (Fig. 3D, Table S1). Non-inoculated fruits (0 hpi) had a total of 1600 and 1360 up- and down-regulated, respectively (Table S2). At 12 and 24 hpi, R showed 1404 and 1496 upregulated genes compared with the S, while 1700 and 1822 were downregulated, respectively. At 48 hpi, the number of DEGs decreased (740 upregulated and 1435 downregulated), but increased again at 96 hpi (1304 upregulated and 2247 downregulated).

The enriched Gene Ontology (GO) Biological Process (BP) terms for up- and down-regulated genes at each time point were summarized in Fig. 3E and detailed in Table S3. Early responses (12–24 hpi) in the resistant genotype showed upregulation of wax and fatty acid biosynthesis related-genes, indicating enhanced cuticle assembly and barrier reinforcement. At the same time, downregulation of genes associated with polysaccharide metabolism and cell wall organization suggested temporary suppression of growth-related remodeling. At 48 hpi, enrichment of phenylpropanoid and secondary metabolite biosynthetic processes reflected activation of inducible defense responses. By 96 hpi, upregulation of signaling and transport pathways indicated hormone-mediated metabolic reprogramming. Together, these patterns illustrate a coordinated transition from early structural fortification to later metabolic and signaling responses.

### Temporal dynamics of DEGs during *B. cinerea* inoculation

**Figure 4:**
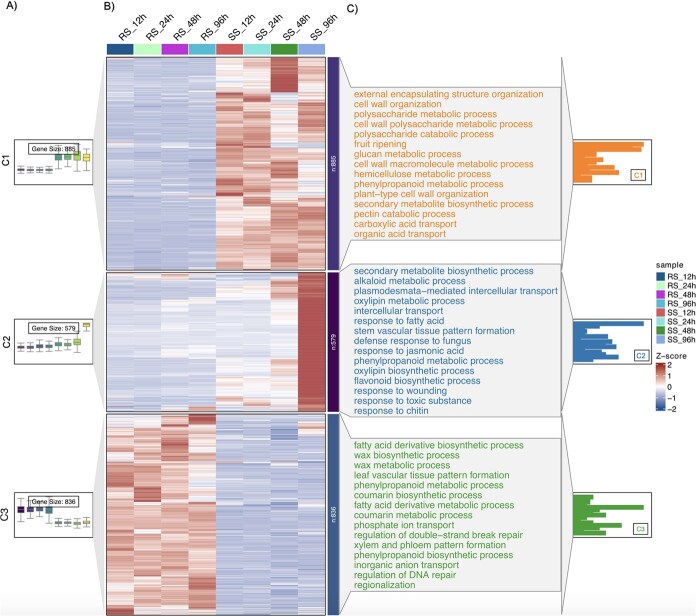
Co-expression clustering of differentially expressed genes in the resistant genotype (RS) compared with susceptible genotype (SS) across time points following *B. cinerea* infection. (A) Boxplots showing the number of DEGs per sample within each co-expression cluster (C1–C3). Boxes summarize gene counts across all samples. (B) Heatmap of DEGs grouped by co-expression clusters, with expression values shown as *Z*-score normalized per gene. Columns represent sample groups corresponding to resistant and susceptible genotypes at 12, 24, 48, and 96 hpi. (C) Top 15 enriched GO BP terms for each cluster. Bar length indicates the enrichment significance (−log₁₀ *P*-value) of each GO term.

To evaluate global gene expression patterns during *B. cinerea* infection, we applied the time-series approach to the DEGs identified. A total of 2300 DEGs were assigned to three clusters (Fig. 4).

Cluster 1 (C1) consisted of 885 DEGs. In S, these genes were high expressed as early as 12 hpi and remained elevated throughout the time course, with moderate fluctuations at 24, 48, and 96 hpi. In contrast, their expression in R remained consistently low. GO enrichment indicated processes associated with cell wall metabolism and remodeling, including polysaccharide metabolic and catabolic processes, cell wall organization, hemicellulose, and pectin catabolism. Additional GO terms included phenylpropanoid and secondary metabolite biosynthetic processes, fruit ripening, and organic acid transport. These results suggest that susceptibility is associated with early and extensive cell wall modifying and metabolic pathways that may facilitate pathogen penetration and colonization.

Cluster 2 (C2) contained 875 DEGs. In S, expression of these genes increased at 48 hpi and peaked at 96 hpi, whereas in R, expression remained relatively stable across timepoints. GO enrichment indicated overrepresentation of secondary metabolic processes, including alkaloid, oxylipin, and phenylpropanoid metabolism, and responses to jasmonic and fatty acids. Enrichment for defense response to fungus and plasmodesmata-mediated intercellular transport suggests that susceptible plants activate these defenses and signaling pathways only at later stages of infection.

Cluster 3 (C3) contained 846 DEGs, with overall expression levels higher in R than in S over the infection time course. GO enrichment analysis showed these genes were associated with fatty acid derivative, wax, phenylpropanoid, coumarin biosynthetic and metabolic processes, as well as leaf vascular tissue pattern formation. Additional enrichment for ion transport, DNA repair, and vascular tissue development suggests that resistance in R involves reinforcement of structural barriers and maintenance of cellular integrity during infection.

### Candidate genes identification through GWAS-transcriptomics integration

Post GWAS, we extracted putative protein-coding genes within ±100 kb of each significant SNP marker (Fig. 2B). Across these intervals, 105 protein-coding genes were identified (Table S4). Intersecting these regions with RNA-seq DEGs revealed eight genes supported by both genetic and transcriptional evidence (Table 3). The expression profiles of these candidate genes and their corresponding positions within the GWAS intervals are shown in Fig. 2B and Fig. 2C. Among them, only *VaccDscaff11-snap-gene-330.64* was upregulated in the resistant genotype, encoding an Importin-13B-like protein. All remaining candidate genes were downregulated in R compared to S. These included *VaccDscaff2-processed-gene-259.6* (DNA ligase 1), *VaccDscaff13-augustus-gene-55.27* (Triacylglycerol lipase 2, *TGL2*), *VaccDscaff13-augustus-gene-55.29* (Myosin-H Heavy Chain-like/EEIG1/EHBP1 protein), *VaccDscaff20-snap-gene-72.61* (L-type lectin-domain containing receptor kinase, LecRK), *VaccDscaff20-snap-gene-39.32* (protein phosphatase 2C, PP2C), *VaccDscaff20-augustus-gene-70.26* (UDP-galactose/UDP-glucose transporter), and *VaccDscaff13-processed-gene-56.14* (uncharacterized protein). Their potential roles in defense and stress response are further described in the Discussion section.

**Table 3:** Differentially expressed genes that are within ±100 kb of significant SNPs associated with DI and fruit DP in blueberries following inoculation with *B. cinerea*.

**Chr**	**Scaffold**	**Marker**	**Start**	**End**	**Gene ID**	**R vs S**	**Timepoint**	**Annotation**
6	VaccDscaff11	33 079 465	33 041 883	33 050 956	*VaccDscaff11-snap-gene-330.64*	UP	48	Importin-13B like
2	VaccDscaff2	25 956 608	25 966 970	25 968 900	*VaccDscaff2-processed-gene-259.6*	DOWN	96	DNA ligase 1
8	VaccDscaff13	5 518 446	5 517 376	5 522 266	*VaccDscaff13-augustus-gene-55.27*	DOWN	96	Triacylglycerol lipase 2 (TGL2)
8	VaccDscaff13	5 518 446	5 554 128	5 561 449	*VaccDscaff13-augustus-gene-55.29*	DOWN	12	Myosin-H Heavy Chain-like/EEIG1/EHBP1 protein
10	VaccDscaff20	7 115 760	7 160 947	7 170 212	*VaccDscaff20-snap-gene-72.61*	DOWN	24	L-type lectin-domain containing receptor kinase (LecRK)
10	VaccDscaff20	3 954 685	3 966 870	3 969 445	*VaccDscaff20-snap-gene-39.32*	DOWN	96	Phosphatase 2C 25 (PP2C)
10	VaccDscaff20	7 115 760	7 040 464	7 044 601	*VaccDscaff20-augustus-gene-70.26*	DOWN	24	UDP-galactose/UDP-glucose transporter 4-like
8	VaccDscaff13	5 518 446	5 599 662	5 604 109	*VaccDscaff13-processed-gene-56.14*	DOWN	12, 24	Uncharacterized protein

Chr, chromosome; R, resistant; S, susceptible; UP, up-regulated; DOWN, down-regulated.

## Discussion

The successful development of blueberries resistant to *B. cinerea* is contingent upon identifying sources of resistance and elucidating the genetic determinants that govern variability in disease outcomes within breeding populations. Despite its significance and impact in postharvest losses, the genetic and molecular underpinnings of fruit rot resistance in blueberries have not been explored so far. Addressing this gap, our research involved a high-throughput screening of the response of 354 southern highbush blueberry genotypes to *B. cinerea* inoculation in fruits. Additionally, we conducted GWAS analyses to identify molecular markers associated with resistance against this pathogen. A complementary time-series RNA-seq analysis was also performed to characterize infection-stage gene expression dynamics between resistant and susceptible genotypes during *B. cinerea* infection.

Investigating genetic mechanisms controlling resistance to pathogens typically starts with the screening of large populations which requires efficient, high-throughput screening protocols to identify promising plant materials for breeding programs [40]. Our protocol allowed for the concurrent screening of hundreds of genotypes using fully mature berries, facilitating an efficient assessment of disease response within a relatively short timeframe of ~2 weeks. This uncovered considerable genetic diversity within our blueberry breeding populations that can be leveraged to enhance resistance to *B. cinerea*.

Our results demonstrated a predominance of additive over non-additive genetic effects across all three disease-related traits. Moderate narrow sense heritability to *Botrytis* resistance has also been reported in other crop species such as strawberry [34] and Arabidopsis [31]. These results demonstrate that genetic factors contribute meaningfully to the observed variation, indicating that progress in population improvement can be achieved through breeding cycles.

Through GWAS and transcriptomic analyses, our study elucidated the genetic and molecular basis of resistance against this pathogen in blueberries. We identified seven significant marker-trait associations, each contributing to a small portion of the phenotypic variance. Our findings support the prevailing view in the literature that resistance to *B. cinerea* is quantitative and involves multiple genes of small effects [31, 41, 42]. Among the candidate genes identified within significant GWAS regions, eight were also differentially expressed in RNA-seq analyses comparing resistant (R) and susceptible (S) genotypes at different time points. Most of these eight candidate genes were downregulated in the R genotype, suggesting that resistance to *B. cinerea* may involve the repression of pathways that necrotrophic pathogens exploit to promote infection. Tight regulation of ABA signaling, lipid metabolism, and cytoskeletal dynamics could prevent excessive cell death and resource diversion, both of which favor *Botrytis* colonization. Thus, R appears to rely on controlled modulation of defense and stress pathways to restrict pathogen progression.

These candidate genes displayed diverse functional annotations, indicating that resistance may involve multiple molecular processes rather than a single defense mechanism. Their functions include intracellular transport, hormone and signal transduction, carbohydrate metabolism, and lipid remodeling- processes commonly associated with plant immune responses. The upregulation of ‘Importin-13B-like gene’ (*VaccDscaff11-snap-gene-330.64*) in R suggests a potential role in nuclear transport and defense signaling, consistent with the reported involvement of importins in stress-related signaling [43]. In contrast, several genes predominantly induced in S, including *VcPP2C* (*VaccDscaff20-snap-gene-39.32*), UDP-sugar transporter (*VaccDscaff20-augustus-gene-70.26*), and *VcLecRK* (*VaccDscaff20-snap-gene-72.61*), are associated with hormone regulation and cell wall remodeling. *PP2C* encodes a negative regulator of the ABA signaling pathway, which modulates MAPK cascades and hormone crosstalk during defense [44–46]. Its lower expression in the resistant genotype may indicate tighter control of ABA-mediated responses, preventing overactivation of pathways that necrotrophic pathogens can exploit. Reduced expression of the *UDP-galactose/UDP-glucose transporter* could also affect cell wall composition and remodeling, consistent with the role of carbohydrate transport in maintaining cell wall integrity during pathogen attack [47]. Moreover, the downregulation of *VaccDscaff2-processed-gene-259.6* related to DNA repair (*DNA ligase 1*) may reflect reallocation of cellular resources from growth and maintenance toward defense. Such transcriptional adjustments are consistent with the cellular remodeling observed during plant–pathogen interactions [48, 49]. Interestingly, *TGL2* (*VaccDscaff13-augustus-gene-55.27*) was strongly upregulated in the susceptible genotype, suggesting enhanced lipid catabolism and membrane turnover under infection. Increased *TGL2* expression may reflect stress-induced mobilization of storage lipids, leading to membrane destabilization and reduced cuticular integrity, conditions favorable for *Botrytis* colonization [50]. While lipase-derived fatty acids can contribute to jasmonate biosynthesis, their expression in the susceptible genotype likely represents a secondary stress response rather than an effective defense mechanism [51]. Collectively, these results suggest that *B. cinerea* resistance in blueberry involves modulation of multiple intracellular and signaling processes that together enhance defense efficiency and limit pathogen progression.

Plant defense against *B. cinerea* relied on both preformed barriers and inducible immune responses. The cuticle, composed of waxes and antifungal compounds, forms the first line of defense by limiting pathogen entry and spore germination as well as sensitive sensors for the timely activation of defense responses [18]. Beneath the cuticle, the plant cell wall acts as a structural barrier, reinforced with cellulose, hemicellulose, pectin, and lignin, which restricts pathogen penetration [19, 20]. From the differential expression and temporal clustering analysis, the R genotype showed enrichment of wax and cuticle biosynthetic processes at early time points, suggesting that reinforcement of the outer protective layers is an important feature of the resistant response. Consistently, correlation analysis revealed a negative association between wax bloom score and DI, indicating that berries with higher wax accumulation were less susceptible to *B. cinerea*. Similar findings have been reported in *Arabidopsis* and tomato, where enhanced wax deposition strengthened cuticular barriers and improved resistance to *B. cinerea* [18, 52] This observation is further supported by previous studies, where increased cuticular wax limited pathogen adhesion and infection in grapes [53] and blueberries [54]. In blueberries, Jiang *et al*. [54] reported that *B. cinerea*-infected berries displayed higher total wax content and upregulation of wax- and cuticle-related genes compared with controls, reinforcing the hypothesis that activation of cuticle biosynthesis contributes to resistance [54]. Moreover, ursolic acid, a major constituent of blueberry cuticular wax and one of several triterpenoid acids, has been shown to inhibit *B. cinerea* spore germination and disrupting fungal cell membrane integrity [52]. Together, these findings emphasize the potential role of wax accumulation as a physical defense barrier against *B. cinerea* infection in blueberry fruits. Future comparative studies of cuticle composition and structure between these genotypes could provide deeper insights into the mechanisms underlying differential defense responses to *B. cinerea*.

In addition to structural barriers, redox and hormone-mediated signaling also contribute to the contrasting responses observed between genotypes. ROS serve as central regulators that integrate environmental signals with defense pathways, coordinating immune activation, hormone crosstalk, and stress adaptation [55–57]. The enrichment of MAPK cascades along with JA- and ET-dependent signaling in the resistant genotype at early time points suggests a timely pathogen recognition. In contrast, delayed expression of these pathways, early ROS activation, and response to SA in the susceptible genotype may reflect disrupted immune coordination. Because necrotrophic pathogens such as *B. cinerea* benefit from host cell death, imbalances in ROS production and hormone signaling could facilitate tissue collapse and colonization [58].

It has been demonstrated in other plant species that *Botrytis* invasion alters cell wall composition, and that cell walls can contribute to either resistance or susceptibility depending on the host response [59]. In cucumber, Polygalacturonase Inhibitor Protein 2 (CsPGIP2) was shown to restrict *Botrytis* growth and colonization [60]. Similarly, knocking out cell wall–remodeling genes such as XTH family members in tomato increased fruit firmness, prolonged shelf life, and reduced *Botrytis* susceptibility [61]. In blueberry, we observed an early and sustained induction of cell wall–modifying genes in the susceptible genotype, that could be leading to active cell wall loosening and metabolic reprogramming during infection. This likely reflects host responses that weaken structural barriers, release nutrients, and reduce apoplastic defenses, thereby facilitating *Botrytis* penetration and colonization. In contrast, the R genotype maintains low expression of these genes, suggesting that prevention of cell wall modifications could contribute to resistance.

Overall, coupling GWAS with time-course RNA-seq, we characterize gray mold fruit disease response in blueberry as a complex trait with small-effect loci and multiple molecular pathways associated with disease-related traits. The overlap of DEGs with GWAS intervals provides a short list of candidates that are supported by both genetic association and genotype-specific expression upon pathogen inoculation, moving beyond locus discovery to mechanism. Transcriptional programs that reinforce cuticle/wax and cell-wall barriers can potentially scale to more resistant genotypes. For breeding purposes, the moderate heritability estimates indicate that breeding gains are achievable; but given its complex genetic architecture, genome-wide selection is likely more effective than phenotypic- or marker-assisted selection alone. Fundamentally, genomic selection captures the cumulative effects of many small-effect loci across the genome, making it more efficient for improving quantitative traits [62], and it has already been successfully applied in blueberry breeding schemes for key fruit-quality traits [63]. Integrating genomic tools with the rapid disease screening assay to train prediction models should speed the culling of susceptible parents and the deployment of cultivars with improved performance under *B. cinerea* infection. In addition, future studies incorporating genotype-by-environment analyses will be critical to assess the stability and durability of resistance under field conditions. Building on the integrative approach of GWAS signals and gene expression responses, this study delivers a set of candidate genes and pathways with supporting evidence that can be prioritized in downstream functional assays and gene editing experiments to confirm their roles and further enhance fruit rot resistance within blueberry breeding germplasm.

## Conclusion

Our study dissected blueberry response to *B. cinerea* by coupling a screening assay with genomic and transcriptomic analyses. We screened 354 blueberry genotypes from two breeding cycles, estimated moderate heritability for disease-related traits, and mapped DI and fruit DP to small-effect loci across several chromosomes, consistent with a quantitative genetic architecture. RNA-seq in resistant and susceptible genotypes, together with GWAS overlap, prioritized eight candidate genes with diverse biological functions. In addition, time-series RNA-seq analyses revealed that, at early time points, the resistant genotype upregulated genes associated with wax and cutin biosynthesis, as well as responses to wounding and fungal-derived molecules, MAPK cascade, ET and JA hormone response suggesting that restricting pathogen penetration and timely pathogen recognition are key components of resistance. This interpretation is further supported by the negative correlation observed at the population level between DI and wax bloom scores. In contrast, the susceptible genotypes exhibited a delayed activation of these defense-related pathways and altered cell wall-related processes that may facilitate pathogen invasion and colonization. The mechanisms identified here represent components of the of basal immune responses triggered by pathogen recognition in the resistant genotype, and some potential susceptibility factors in the susceptible interaction. Considerable overlap exists among this canonical plant defense mechanism across different pathogen types, particularly those for broad-range necrotrophic pathogens, such as *Botrytis*. Understanding how these shared immune layers contribute to resistance to other pathogens may facilitate the development of durable and broad-spectrum strategies for resistance breeding. Taken together, these findings shed light on the genetic basis of resistance against *B. cinerea* in blueberries presenting promising avenues for future breeding efforts in this crop.

## Materials and methods

### Experimental material

Phenotyping was conducted on 354 advanced selections of southern highbush blueberry from the Blueberry Breeding Program at the University of Florida. Test plots were located in Waldo, FL (USA), and managed under standard commercial production practices. Genotypes originally came from two breeding cycle populations (Popn-I and Popn-II) that were generated from distinct parental combinations and selection cycles. Popn-I comprised 205 genotypes assessed for response to *B. cinerea* inoculation in May 2022; Popn-II comprised 149 genotypes evaluated in May 2023. To account for inter-year effects, 48 genotypes from Popn-I were re-included as reference checks during the Popn-II evaluation (Fig. 1A). For each genotype, 60 fully ripe and uniform berries, free of any visible mechanical injury and pests or disease, were harvested and used for phenotyping. Out of the 60 total berries collected at harvest, 30 berries per genotype were allocated for spray inoculation to assess disease response, while the remaining 30 berries were reserved for fruit quality analysis. For disease screening assay, prior to inoculation, 10 berries were assorted into each of the 3 separate clamshells, forming a total of 3 replicates per genotype.

### Source of isolate and preparation of inoculum

A monoconidial isolate of *B. cinerea* was used to prepare the inoculum for both experimental setups. The isolate, originally collected by Dr Philip Harmon’s group at the University of Florida, from naturally infected blueberries under postharvest storage in Florida, was maintained on potato dextrose agar (PDA) and incubated at 25°C under continuous fluorescent lighting for 7 days. Conidia were counted using a hemacytometer (Bright-Line Hemacytometer; Hausser Scientific, Horsham, PA, USA), and the suspension was adjusted to 1 × 10^6^ conidia ml^−1^ in autoclaved distilled water for spray application.

### Inoculation procedure

Berries within each clamshell were uniformly coated with 1 × 10^6^ conidia ml^−1^ suspension of *B. cinerea* until runoff, using an air pen brush (Paasche VL-SET Double Action Siphon Feed Airbrush, Paasche Airbrush Company, Chicago, IL, USA). For negative controls, ten berries from each of four selected genotypes were placed in separate clamshells using the same replicate structure and sprayed with sterile distilled water.

Following inoculation, clamshells were placed in sealed plastic containers lined with filter paper moistened with sterilized distilled water to maintain ~85%–90% relative humidity (RH) and incubated at 23°C for 3 days [54]. To promote *B. cinerea* sporulation while suppressing *Colletotrichum* (anthracnose) overgrowth, clamshells were then transferred to growth chambers at 10°C and ~95% RH for 5 days (conditions known to enhance gray mold development while disfavoring *Colletotrichum* sporulation) [64–66]. After this phase, berries were returned to 23°C under the initial RH conditions to facilitate rapid disease assessment. Disease response was monitored through 16 dpi (Fig. S1).

Additionally, to confirm *B. cinerea* infection, berry skin tissue was excised from fifteen randomly selected genotypes, together with uninoculated controls. Tissues were surface sterilized in 10% (v/v) sodium hypochlorite for 1 min, rinsed three times in sterile water (1 min each), plated on PDA, and incubated at 25°C under continuous fluorescent light for 7 days. Conidial growth with morphology consistent with the reference *B. cinerea* isolate was recovered from all inoculated samples (symptomatic and asymptomatic), but not from water control samples.

### Disease phenotyping

Three disease-related traits were visually scored in this study: (i) days to the appearance of first mycelium or DFM; (ii) count/incidence of infected berries out of 10 berries, termed as disease incidence or DI; and (iii) the spread of mycelia on the fruit surface causing decay as fruit decay percentage or DP. The genotypes were monitored daily until the end of the experiment to record DFM in each of the clamshells. DI and DP for every clamshell were recorded at the end of 16 dpi. The phenotypic data score for the three disease-related traits and replicates are provided in Table S5.

### Fruit quality

Fruit quality parameters, including wax bloom, firmness, size, °Brix, total titratable acidity (TTA), and scar size were assessed on the day of harvest, following the methodology outlined by [67]. Wax bloom scoring, indicative of the wax coating on the fruit’s surface, was rated with a single ordinal score per clamshell based on the visually estimated proportion of epicuticular wax coverage on the fruit surface: 1 = 0%–25%, 2 = 25%–50%, 3 = 50%–75%, and 4 = 75%–100%. Fruit firmness and size were measured using a Fruit Firm 1000 machine (CVM, Inc.)—firmness was quantified by the mean force required to deflect the fruit surface by 1 mm (g/mm), and size was recorded as the fruit diameter in millimeters. The fruit samples were blended and centrifuged, and the obtained juice was evaluated for soluble solids content (°Brix) with a digital pocket refractometer (Atago USA, Inc.) and for TTA with an automatic titrator (Mettler Toledo, Inc.). Pedicel scar size was assessed on a subsample of 10 berries per genotype, categorized as small, medium, or large and summarized through a scar-size coefficient [67]. Fruit quality data is provided in Table S6.

### Genotypic data

Genotyping was performed at RAPID Genomics (Gainesville, FL, USA) using Capture-Seq approach with a genome-wide panel comprising of 10 000 biotinylated ~120-mer probes [63]. Juvenile leaf tissue was sampled from each selection for DNA extraction, followed by target-enriched library preparation and Illumina HiSeq2000 paired-end sequencing (150-cycles).

Raw reads were subjected to quality-based filtering and trimming, then aligned to the largest haploid scaffold assembly of the *V. corymbosum* cv. ‘Draper’ reference genome [68] using Mosaik v.2.2.3 [69]. SNPs were called with the 10 000 probe positions as targets using FreeBayes v.1.3.2 [70]. SNPs were retained if they met all of the following criteria: mapping quality ≥10, biallelic state, missing data ≤50%, and a minor allele frequency ≥0.01. Allele-specific read counts per individual were extracted from the VCF using Vcftools v. 0.1.16 [71]. Allele dosages were inferred from read counts using the updog package v.2.1.0 [72]. Only SNPs accurately genotyped in 95% of the individuals were kept. Dosage was encoded for the alternate allele (B) relative to the reference allele (A) for tetraploids as: 0 (AAAA), 1 (AAAB), 2 (AABB), 3 (ABBB), and 4 (BBBB) [73]. Genotypic classes are provided in Table S7.

### Phenotypic analysis

The datasets from both populations (Popn-I and Popn-II) were combined (Comb_popn) separately for DFM, DI and DP. The best linear unbiased estimates (BLUEs) were estimated for each of the three traits. Notably, for BLUEs estimation, a one-stage correction was applied using the overlapping 48 genotypes as checks and enabling the correction of year-effects [74]. Thus, adjusted means for each genotype were obtained using the following linear model:


$$ {y}_{ij k}=\mu +{B}_i+R/{B}_{ij}+{G}_{ij k}+{e}_{ij k}, $$

where ${y}_{ijk}$ is the phenotype of the disease response (DFM, DI or DP) of genotype *i* in year *j* and replicate *k*; $\mu$ is the overall mean; ${B}_i$ is the fixed year effect; $R/{B}_{ij}$ is the fixed replicate effect under each year; ${G}_{ijk}$ is the genotypic effect of individual *i* at year *j* and replicate *k*; and ${e}_{ijk}$ is the random residual effect assumed to be independent and normally distributed (${e}_{ijk}\sim N\left(0,{\sigma}_e^2\right)\Big)$, where ${\sigma}_e^2$ is the residual variance. For the genotypic effect, the experiment was designed as an Augmented Block Design with common checks connecting both years. Thus, the genotypic effect was separated into two groups, with the fixed effect for the regular individuals and the fixed effect associated with the checks. The BLUEs of each genotype were used as the response variable in subsequent analyses.

Furthermore, heritability estimates were computed to quantify the proportion of additive and non-additive genetic variation attributed to genetic factors by considering genotypes as random effects and incorporating genomic information into the relationship matrix: ${\boldsymbol{y}}^{\ast }=\mathbf{1}\mu +{Z}_a\boldsymbol{a}+{Z}_d\boldsymbol{d}+\boldsymbol{e}$**, w**here ${\boldsymbol{y}}^{\ast }$ is the vector of BLUEs; μ represents the population mean with assumed flat prior distribution; $\boldsymbol{a}$ is the vector of additive genetic effects which follows a normal distribution with $\boldsymbol{a}\sim N\left(\boldsymbol{O},\boldsymbol{G}{\sigma}_a^2\right),$  ${\sigma}_a^2$ is the additive genetic variance and $\boldsymbol{G}$ is the additive (genomic) relationship matrix; d is the vector of non-additive genetic effects which follows a normal distribution with d$\sim N\left(\boldsymbol{O},\boldsymbol{I}{\sigma}_d^2\right),{\sigma}_d^2$ is the non-additive genetic variance and I is an identity matrix; and ${Z}_a$ and ${Z}_d$ are incidence matrix of a and d, respectively; and $\boldsymbol{e}$ is the vector of random errors with $\boldsymbol{e}\sim N\left(\boldsymbol{O},\boldsymbol{I}{\sigma}_e^2\right)$ and ${\sigma}_e^2$ is the residual variance. The construction of the genomic relationship matrix (G) was done using the AGHmatrix package in R assuming tetrasomic inheritance [75]. This model was implemented by the BGLR package in the R software [76], defining 12 000 iterations for the Markov chain Monte Carlo (MCMC), a burn-in period of 2000 MCMC cycles, and a posterior thinning of 5. Variance components were computed as the posterior mean. The narrow sense heritability *(h^2^)* was estimated as: $\left({h}^2\right)=\frac{\sigma_a^2}{\sigma_a^2+{\sigma}_e^2}$ and the broad sense heritability was estimated as $\left({H}^2\right)=\frac{\sigma_a^2+{\sigma}_d^2}{\sigma_a^2+{\sigma}_d^2+{\sigma}_e^2}$.

Additionally, we tested linear associations between several fruit quality and disease traits using Pearson correlations with a significance threshold of *P* < 0.01.

### Genome-wide association study

To identify genomic regions regulating response to *B. cinerea*, we conducted GWAS separately for each of the three traits DFM, DI and DP, using the R package GWASpoly v.2.11, [73]. Population structure was characterized by PCA of the genomic relationship matrix (Fig. S4) and was accounted for by constructing a Q matrix from the top five principal components and calculating kinship matrices (K) using the algorithm within GWASpoly. Associations between SNPs and phenotypic responses were analyzed using a linear mixed model (Q + K), as described by Rosyara *et al*. [73]. We explored four genetic models: general, additive, simplex dominant, and duplex dominant. An adjusted Bonferroni correction set at a significance threshold of 0.05 was used to define the *P*-value detection limit. Associations between SNPs and phenotypes were further validated using quantile-quantile plots, displaying estimated −log10 (*P*) values.

### RNA-seq experiment design and library preparation

Two genotypes with contrasting DI responses to *B. cinerea* were selected from the screening assay: a resistant (‘R’) with no disease symptoms in any fruit and a susceptible (‘S’) with all fruits rotted. For this experiment, ripe and healthy berries were harvested again and inoculated as in the screening assay. For each genotype, three clonally propagated plants were used as biological replicates and 10 fully ripe fruits of each plant were placed in clamshells and kept at high humidity under long-day conditions at 23°C. Fruits were imaged and collected at 12, 24, 48, and 96 hpi. Untreated fruit at 0 h served as baseline controls to capture natural transcriptional variation between genotypes. Samples were flash-frozen in liquid nitrogen and stored at −80°C until RNA extraction.

Total RNA was extracted from each replicate (pool of 10 berries) using the Plant/Fungi RNA Purification Kit (Norgen Biotek, Canada). RNA quantity and integrity were assessed with QUBIT fluorescent method (Invitrogen, USA) and 2100 Bioanalyzer (Agilent Technologies, USA). A total of 500 ng RNA with RNA integrity number > 7 was used as input material for the mRNA stranded library construction. Sequencing libraries were generated using NEBNext® Ultra™ II Directional RNA Library Prep Kit for Illumina® (New England Biolabs, USA) following the manufacturer’s recommendations. RNA-seq libraries were prepared and sequenced (2 × 150 bp) on an Illumina NovaSeq X Plus at the University of Florida Interdisciplinary Center for Biotechnology Research (ICBR). Raw reads were assessed with FastQC v0.11.7 [77] and trimmed with Trim Galore v0.6.10 (https://github.com/FelixKrueger/TrimGalore). Filtered reads were aligned to the *V. corymbosum* ‘Draper’ reference genome (largest haploid chromosome set) with HISAT2 [78], and gene-level read counts were obtained using HTSeq-count [79].

### Principal components and differential gene expression analysis

DEGs were identified using DESeq2 [80] within the R v4.4.1/Bioconductor framework. Pairwise differential expression analyses were performed for the R and S genotypes at each time point. To account for inherent transcriptional differences between genotypes, untreated samples (R₀ vs S₀) were first compared, and genes differentially expressed at this baseline were excluded from subsequent time-point comparisons (Table S4). DEGs were selected based on a false discovery rate threshold of *P* < 0.05 and an absolute fold-change >2.

PCA was conducted using variance-stabilized transformed (VST) expression values obtained from DESeq2 to assess overall transcriptional variation. Genes expressed in at least 25% of samples were retained, and the average VST expression of three biological replicates per genotype and time point was used for visualization with the plotPCA() function in R.

### Candidate genes identification

To identify potential candidate genes linked to *B. cinerea*-associated phenotypes, we integrated the results from GWAS and RNA-seq analyses. Following GWAS, for each trait-associated SNP, a ±100-kb window was defined to search for candidate genes, according to an approximation of linkage disequilibrium decay block estimated in the blueberry breeding population [81]. When significant SNPs were <100 kb apart, we merged them into one interval spanning (leftmost SNP −100 kb) to (rightmost SNP +100 kb). Genes within these intervals were retrieved and functionally annotated with PANNZER2 [82] and eggNOG-mapper [83]. Following transcriptomic analyses, DEGs were selected from pairwise comparisons (R vs S) at each time point and checked for the overlap within the significant GWAS regions.

### Temporal co-expression clustering and functional enrichment analysis

DEGs were clustered using the k-means method in ClusterGVis R package based on their time-series expression patterns. Functional enrichment of both time-point-specific DEG sets and co-expression clusters was performed using the enrichCluster function from the clusterProfiler R package (v4.8.3) [84]. The annotation database (org.Vcorymbosum.eg.db) was created using the GO terms annotation from the cv. ‘Draper’ predicted proteome as the reference for functional annotation. BP terms with adjusted *P* < 0.05 were considered significant, and all significant GO categories were retained for downstream interpretation and visualization. GO terms were summarized using REVIGO [85].

## Supplementary Material

Web_Material_uhag092

## Data Availability

The genotypic and phenotypic datasets are available within the supplementary materials of this paper. RNAseq data is available at the NCBI SRA repository under BioProject accession PRJNA1372075.

## Supplementary material

Supplementary material will be available at *Horticulture Research* online.
