# Supplementary material for: Integrative GWAS and transcriptomic analyses reveal markers and candidate genes associated with resistance to Botrytis cinerea fruit rot in blueberry
Source: Hortic Res. 2026 Mar 13;13(7):uhag092. doi: 10.1093/hr/uhag092 (PMC13271799; doi:10.1093/hr/uhag092)
Supplement: Web_Material_uhag092 [file web_material_uhag092.zip › Supplementary_figures_S1_S4.pdf]

Supplementary Figures

Integrative GWAS and transcriptomic analyses reveal markers and candidate genes associated with resistance to Botrytis cinerea fruit rot in blueberry

Lushan Ghimire1,#, Yichun Wang1,#, Paul Adunola1, Warda Boukari2, Gonzalo Casorzo1, Felix Enciso-Rodriguez1, Philip F. Harmon2, Juliana Benevenuto1 and Patricio R. Munoz1*

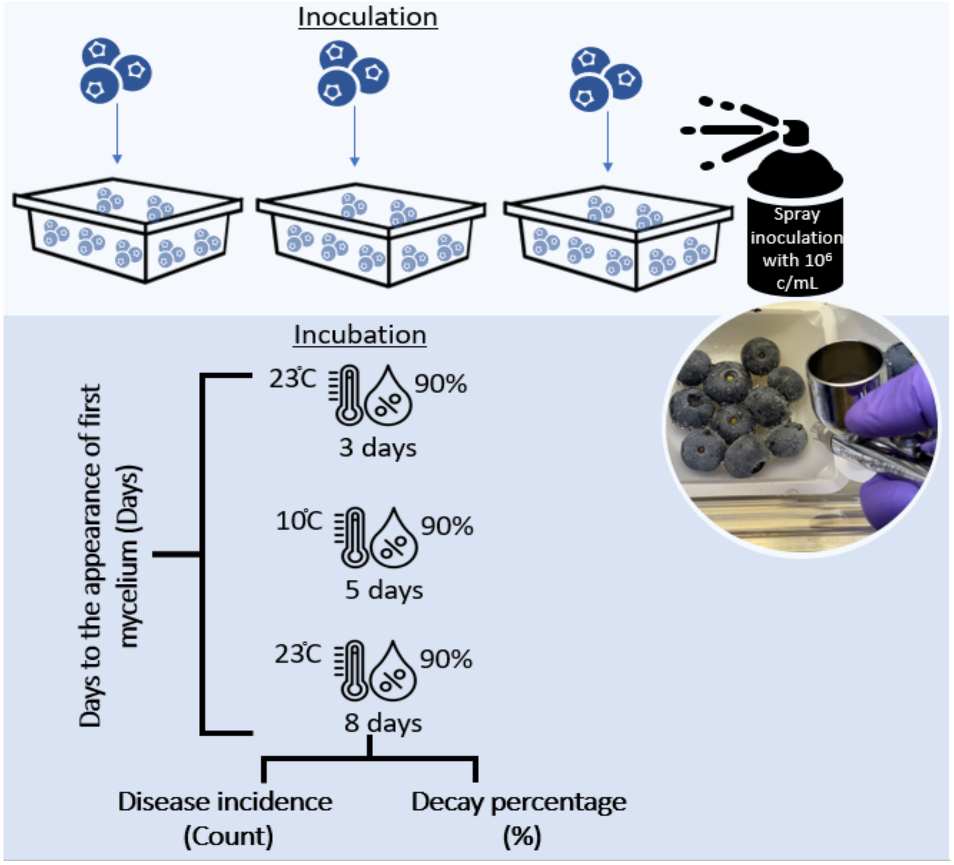

Figure S1: Screening protocol developed to screen blueberry fruits against gray mold caused by *Botrytis cinerea* in large breeding populations.

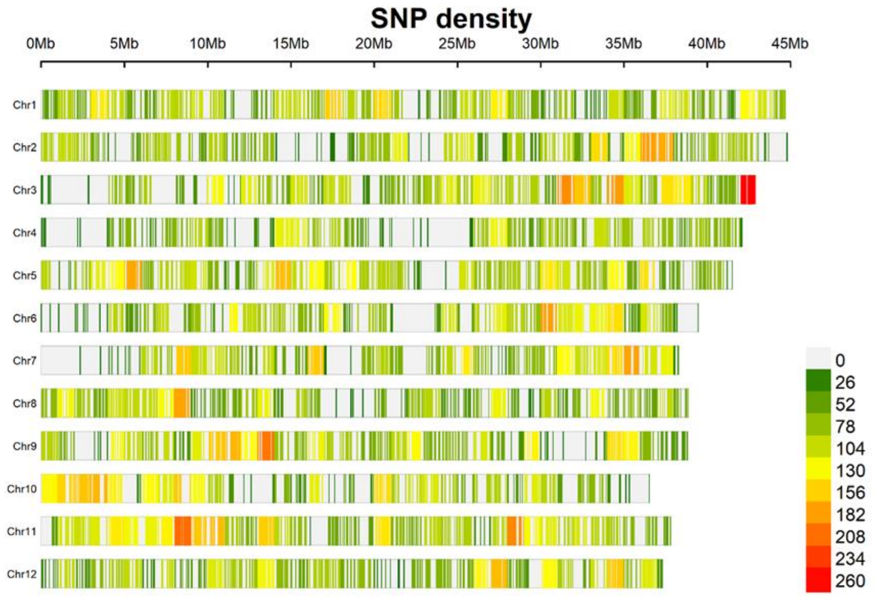

Figure S2: **Figure**

Figure S2: Distribution of 38,379 single nucleotide polymorphisms (SNPs) in 1 Mb window size across the 12 blueberry chromosomes. The x-axis represents the distance in base pairs. The color heatmap represents the number of SNPs.

A)

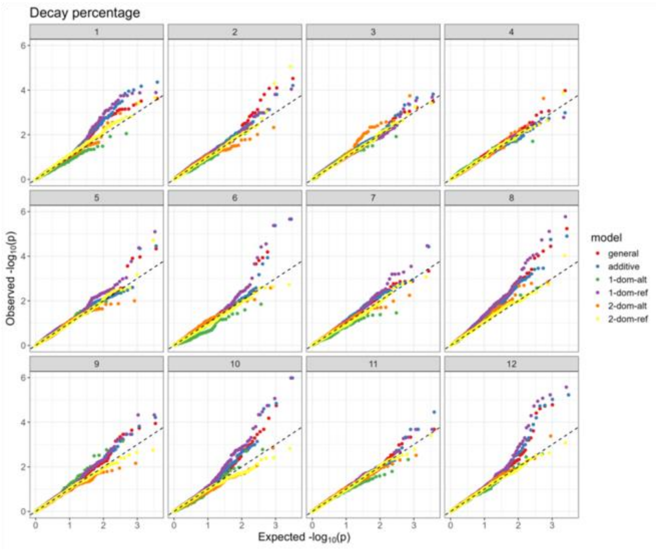

B)

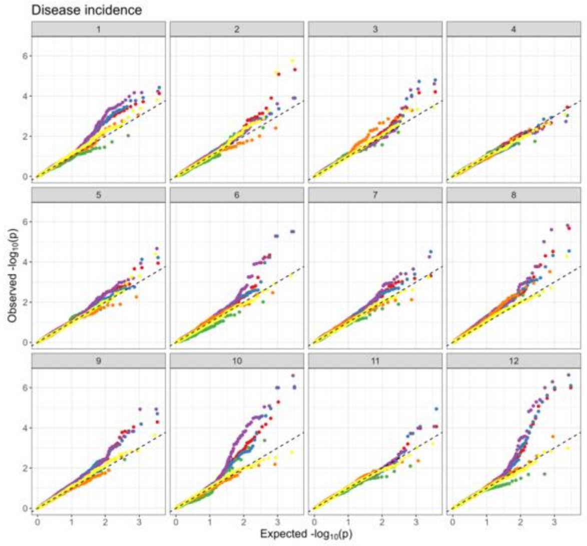

Figure S3: Quantile-quantile (Q-Q) plots of GWAS results for gray mold resistance with several gene action models in the combined southern highbush blueberry population: **A)** Q-Q plot for fruit decay percentage and, **B)** Q-Q plot for disease incidence . The observed association significance is plotted against the expected association significance for each SNP.

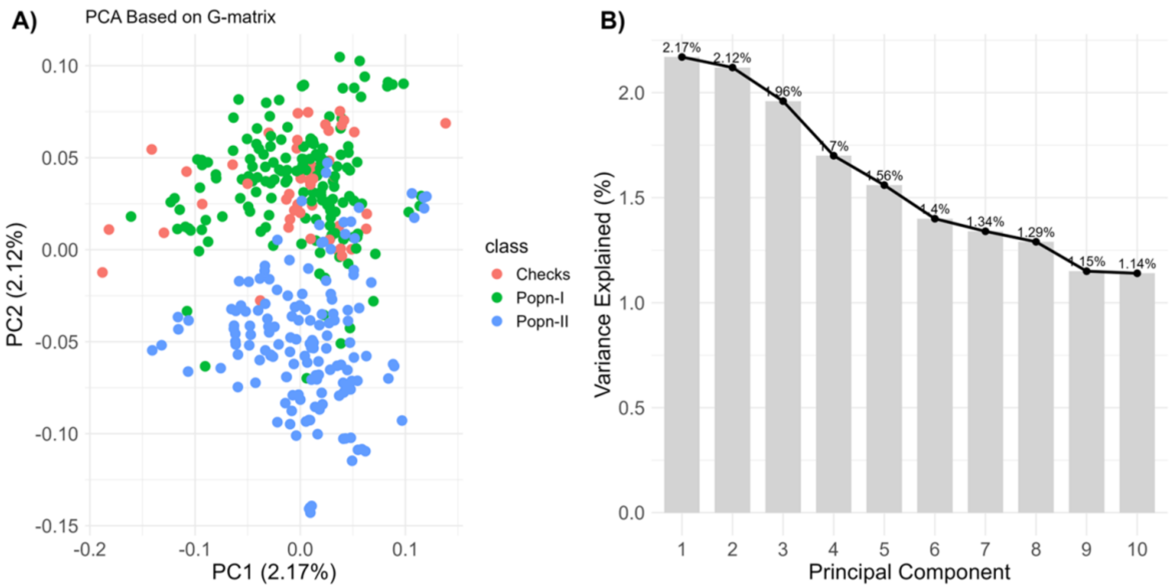

Figure S4: **A)** Genomic PCA based on the SNP-derived genomic relationship matrix of southern highbush blueberries within two different breeding populations (Popn-I and Popn-II), comprising a total of 354 genotypes. Each point represents an individual in the hyperspace defined by the eigenvectors of the first and second principal components. **B)** Scree plot showing the contribution of the first ten principal components to the underlying population structure.
